# Organ-specific metastatic landscape dissects PD-(L)1 blockade efficacy in advanced non-small cell lung cancer: applicability from clinical trials to real-world practice

**DOI:** 10.1186/s12916-022-02315-2

**Published:** 2022-04-12

**Authors:** Si-Cong Ma, Xue Bai, Xue-Jun Guo, Li Liu, Lu-Shan Xiao, Yan Lin, Jia-Le Tan, Xiao-Ting Cai, Yu-Xiang Wen, Hu Ma, Q. John Fu, Meng-Xin Leng, Yan-Pei Zhang, Li-Li Long, Ze-Qin Guo, De-Hua Wu, Jian-Guo Zhou, Zhong-Yi Dong

**Affiliations:** 1 Department of Radiation Oncology, Nanfang Hospital, Southern Medical University, Guangzhou, China; 2 Information Management and Big Data Center, Nanfang Hospital, Southern Medical University, Guangzhou, China; 3 Department of Medical Quality Management, Nanfang Hospital, Southern Medical University, Guangzhou, China; 4 Guangdong Provincial Key Laboratory of Viral Hepatitis Research, Hepatology Unit and Department of Infectious Diseases, Nanfang Hospital, Southern Medical University, Guangzhou, China; 5 Department of Oncology, The Second Affiliated Hospital of Zunyi Medical University, Zunyi, China; 6 Department of Community Health, Tufts University, Medford, USA; 7 Department of Radiation Oncology, Universitätsklinikum Erlangen, Erlangen, Germany; 8 Comprehensive Cancer Center Erlangen-EMN, Erlangen, Germany

**Keywords:** Metastatic-organ landscape, Non-small cell lung cancer, Immune checkpoint inhibitor, Programmed death-(ligand) 1

## Abstract

**Background:**

Organ-specific metastatic context has not been incorporated into the clinical practice of guiding programmed death-(ligand) 1 [PD-(L)1] blockade, due to a lack of understanding of its predictive versus prognostic value. We aim at delineating and then incorporating both the predictive and prognostic effects of the metastatic-organ landscape to dissect PD-(L)1 blockade efficacy in non-small cell lung cancer (NSCLC).

**Methods:**

A total of 2062 NSCLC patients from a double-arm randomized trial (OAK), two immunotherapy trials (FIR, BIRCH), and a real-world cohort (NFyy) were included. The metastatic organs were stratified into two categories based on their treatment-dependent predictive significance versus treatment-independent prognosis. A metastasis-based scoring system (METscore) was developed and validated for guiding PD-(L)1 blockade in clinical trials and real-world practice.

**Results:**

Patients harboring various organ-specific metastases presented significantly different responses to immunotherapy, and those with brain and adrenal gland metastases survived longer than others [overall survival (OS), *p* = 0.0105; progression-free survival (PFS), *p* = 0.0167]. In contrast, survival outcomes were similar in chemotherapy-treated patients regardless of metastatic sites (OS, *p* = 0.3742; PFS, *p* = 0.8242). Intriguingly, the immunotherapeutic predictive significance of the metastatic-organ landscape was specifically presented in PD-L1-positive populations (PD-L1 > 1%). Among them, a paradoxical coexistence of a favorable predictive effect coupled with an unfavorable prognostic effect was observed in metastases to adrenal glands, brain, and liver (category I organs), whereas metastases to bone, pleura, pleural effusion, and mediastinum yielded consistent unfavorable predictive and prognostic effects (category II organs). METscore was capable of integrating both predictive and prognostic effects of the entire landscape and dissected OS outcome of NSCLC patients received PD-(L)1 blockade (*p* < 0.0001) but not chemotherapy (*p* = 0.0805) in the OAK training cohort. Meanwhile, general performance of METscore was first validated in FIR (*p* = 0.0350) and BIRCH (*p* < 0.0001), and then in the real-world NFyy cohort (*p* = 0.0181). Notably, METscore was also applicable to patients received PD-(L)1 blockade as first-line treatment both in the clinical trials (OS, *p* = 0.0087; PFS, *p* = 0.0290) and in the real-world practice (OS, *p* = 0.0182; PFS, *p* = 0.0045).

**Conclusions:**

Organ-specific metastatic landscape served as a potential predictor of immunotherapy, and METscore might enable noninvasive forecast of PD-(L)1 blockade efficacy using baseline radiologic assessments in advanced NSCLC.

**Supplementary Information:**

The online version contains supplementary material available at 10.1186/s12916-022-02315-2.

## Background

Immune checkpoint inhibitors (ICIs), principally represented by monoclonal antibodies targeting programmed death-1 (PD-1) or programmed death-ligand 1 (PD-L1), have transformed the therapeutic paradigm and become the pillar regimens for advanced or metastatic non-small cell lung cancer (NSCLC) [1, 2]. Despite the relative success of immunotherapy over chemotherapy witnessed in the past decade, PD-(L)1 blockade was far from perfect to induce durable immune responses for every patient from the total unselected NSCLC population, even within the PD-L1-positive stratum, calling for an exigent need of reliable immunotherapeutic predictors to promote precision medicine.

Effectiveness of ICI therapy has been associated with the biological peculiarity of tumor microenvironment differed by anatomical locations [3]. By a logical extension of this notion, it is envisioned that the heterogeneity of immunotherapeutic efficacy in the metastatic setting might depend on the interplay of the overall antitumor immunity with the local metastatic-organ microenvironment [4]. Preliminary evidences have demonstrated the negative correlation between survival prospects and distant metastases (e.g., liver metastasis) in NSCLC treated with ICIs [5, 6]. Nevertheless, these studies were conducted in a single-arm setting of ICIs, which lacked a controlled arm of conventional treatment for comparison, resulting in an ignorance of the predictive versus prognostic effect [7]. Besides, the therapeutic impact of each metastatic organ was previously investigated in isolation, but comprehensive analysis that looks at the big picture and incorporates the entire metastatic landscape is still lacking; as tumors could spread to several organs concurrently for patients with advanced stage cancers, outcome studies that focused on single organ metastasis presented limited clinical significance.

On this ground, we assumed that only by integrative delineation of the whole metastatic-organ landscape from both perspectives of prognostic and predictive effects could we systematically assess the impact of organ metastases on ICI therapy. For this purpose, we discriminated the predictive versus prognostic effect of each metastatic organ within a double-arm randomized trial cohort, and further proposed a metastasis-based scoring system (METscore), which incorporated both prognostic and predictive effects of the metastatic-organ landscape, to forecast survival outcomes of advanced NSCLC patients treated with ICI therapy in clinical-trial and real-word cohorts.

## Methods

### Population

We conducted a retrospective study of individual-participant data from three clinical trials (OAK: NCT02008227, *N* = 850; FIR: NCT01846416, *N* = 136; BIRCH: NCT02031458, *N* = 667) [8–10] and a real-word cohort from Nanfang Hospital, Southern Medical University (NFyy: *N* = 409), giving a total sample size of 2062 advanced-stage NSCLC patients. OAK was a randomized phase III trial comparing atezolizumab versus docetaxel in previously treated NSCLC patients [11, 12]. FIR and BIRCH were both phase II atezolizumab trials in chemotherapy-naïve or previously treated PD-L1-selected NSCLC patients [13, 14]. Detailed information about these three clinical trials has been described in the corresponding studies [11, 13, 14]. Deidentified individual-participant data of the clinical trials were accessed according to Roche’s policy and process for clinical study data sharing. The NFyy cohort included patients diagnosed with metastatic NSCLC who were treated with at least one dose of ICIs as first-line or subsequent regimen, which was collected after the approval of the Institutional Ethical Review Boards of Nanfang Hospital. The clinical characteristics of the NFyy cohort are summarized in Additional file 1: Table S1.

Baseline PD-L1 status of the three trial cohorts was prospectively tested using the SP142 antibody, scored as a percentage of tumor cells (TC0: < 1%; TC1: ≥ 1% but < 5%; TC2: ≥ 5% but < 50%; TC3: ≥ 50%) or tumor-infiltrating immune cells (IC0: < 1%; IC1: ≥ 1% but < 5%; IC2: ≥ 5% but < 10%; IC3: ≥ 10%) with PD-L1 staining [11, 13, 14]. PD-L1 expression of the real-world NFyy cohort was tested before treatment using the 22C3 antibody and calculated as a tumor proportion score (TPS). Patients with PD-L1 TC/IC or TPS of at least 1% were considered positive for the respective immunohistochemistry assays. Among these cohorts, 465 patients in OAK and 367 in NFyy were tested as PD-L1 positive, while patients in PD-L1-selected FIR and BIRCH trials were all confirmed positive at enrollment.

### Study design

**Fig. 1:**
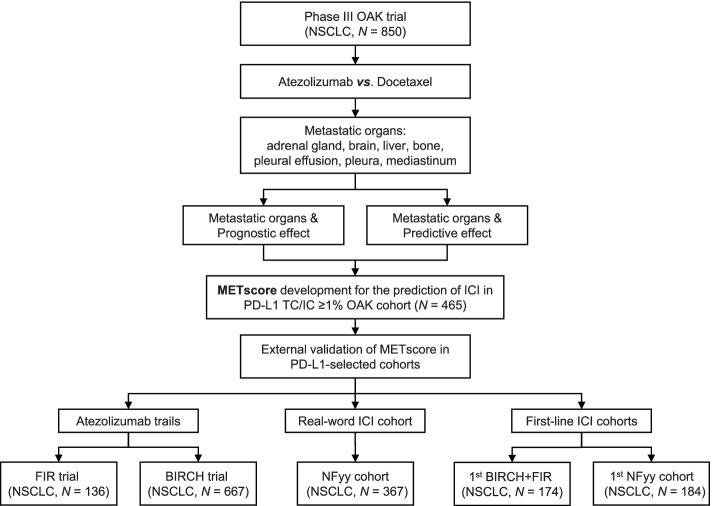
Flowchart showing the design of the study. The double-arm OAK trial (atezolizumab versus docetaxel) was used as a discovery cohort to identify the prognostic and predictive effects of metastatic organs. A metastasis-based scoring system, termed METscore, was developed to forecast survival prospects of ICI therapy in the PD-L1-positvive population, and was further validated in PD-L1-selected atezolizumab trials (FIR and BIRCH) and a real-word ICI cohort. Abbreviations: NSCLC, non-small cell lung cancer; ICI, immune checkpoint inhibitor; PD-L1, programmed death-ligand 1; TC, tumor cell, IC: immune cell

The workflow of the study was illustrated in Fig. 1. We first took advantage of the OAK trial as it endorsed two arms of patients (immunotherapy versus chemotherapy) so that we were able to delineate the predictive versus prognostic effect of distant metastases. Then, a metastasis-based scoring system, termed METscore, was developed to incorporate both the immunotherapy-specific predictive effect and the treatment-independent prognostic effect of the whole metastatic-organ landscape, thereby forecasting clinical outcomes of ICI therapy. The generalization performance of METscore was tested in single-arm FIR and BIRCH trials and the real-world NFyy cohort. Moreover, the discrimination ability was validated in the first-line ICI setting. Overall survival (OS) was defined as the primary endpoint, and progression-free survival (PFS) was the co-primary endpoint as an addition to OS for first-line cohorts.

### Prognostic and predictive effects of metastatic-organ landscape

Pretreatment statuses of common distant metastatic organs of NSCLC were included for analysis; those with incidence of < 5% in OAK were not examined. Thus, the metastatic-organ landscape comprised adrenal glands, brain, liver, bone, pleura, pleural effusion, and mediastinum in the present study. These metastatic organs were divided into two categories based on their predictive value for ICI therapy. Accordingly, category I metastatic organs were defined as those with survival benefits of immunotherapy over chemotherapy, while others were defined as category II.

Multivariate Cox proportional hazard regression was performed to delineate the predictive versus prognostic effect of each metastatic organ via the inclusion of metastatic statuses of organs, treatment arm, and treatment-by-organ interaction terms using the randomized OAK data (Additional file 2: Table S2). The model coefficients were scaled from − 5 to 5 and rounded to the nearest integers to facilitate clinical use [15, 16]. The scaled points corresponding to the metastatic statuses of organs delineated the treatment-independent prognostic effects, while those corresponding to treatment-by-organ interactions delineated the immunotherapy-specific predictive effects (Additional file 3: Table S3). The METscore in forecasting outcomes of ICI therapy was then set up to incorporate the prognostic and predictive effects of each metastatic organ by calculating the total points.

### Statistical analysis

Kaplan-Meier method and log-rank test were used for comparing survival outcomes among groups, with hazard ratio (HR) and 95% confidence interval (CI) reported. Survival analyses were conducted in GraphPad Prism (version 8.0.1) or R (version 3.6.1) with R packages “survival” and “survminer.” Multivariate Cox proportional hazard regression was implemented using R package “survival.” The optimum cut-point for METscore was determined by maximally selected rank statistics using the R package “maxstat” with a minimal proportion of 20% in each group by setting the parameter “minprop” as 0.2. All statistical tests were two sided, and *P* ≤ 0.05 was deemed statistically significant.

## Results

### Specific predictive effect of metastatic-organ landscape for ICI therapy

**Fig. 2:**
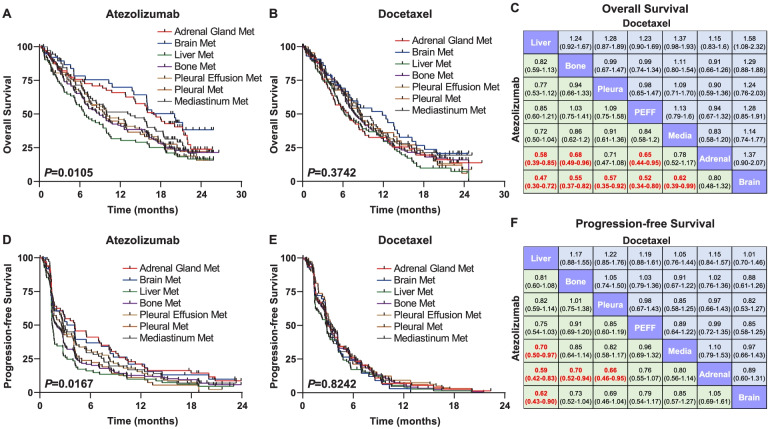
Metastatic-organ landscape as a determinant of survival outcomes specific to immune checkpoint-based therapy. Kaplan-Meier curves comparing overall survival among various metastatic organs in **A** atezolizumab- and **B** docetaxel-treated populations. **C** Pairwise comparisons of overall survival in the atezolizumab- (lower triangle) and docetaxel-treated (upper triangle) populations. Kaplan-Meier curves comparing progression-free survival per metastatic organs in **C** atezolizumab- and **D** docetaxel-treated populations. **E** Pairwise comparisons of progression-free survival in the atezolizumab- (lower triangle) and docetaxel-treated (upper triangle) populations. Data in each cell of **C** and **F** represents hazard ratio (95% interval confidence) for the pairwise comparison of row-defining organ versus column-defining organ. Significant results are in bold red font. Abbreviations: Met, metastasis; PEFF, pleural effusion; Media, mediastinum

To delineate the immunotherapeutic predictive effect of organ metastases, we explored the impact of metastatic-organ landscape on survival outcomes in the atezolizumab arm and the docetaxel arm from OAK respectively. In terms of OS, survival prospects were significantly different among patients with various metastatic organs in the atezolizumab-treated population (*P* = 0.0105; Fig. 2A). Nevertheless, OS was generally similar in the docetaxel-treated population regardless of metastatic organs (*P* = 0.3742; Fig. 2B). Pairwise comparisons among metastatic organs in the atezolizumab arm showed that adrenal gland metastasis and brain metastasis yielded the best long-term survival benefits, with significantly longer OS compared with metastases to other organs, including liver, bone, and pleural effusion (the lower triangle in Fig. 2C). With regard to the docetaxel arm, however, no significant difference of OS existed between any pair of metastatic organs (the upper triangle in Fig. 2C).

Consistent findings were observed in terms of PFS, where significant survival difference was seen among atezolizumab-treated patients with various metastatic organs (*P* = 0.0167; Fig. 2D) but not in docetaxel-treated populations (*P* = 0.8242; Fig. 2E). In agreement with this, there only existed significant differences of PFS in pair-wise comparisons in the atezolizumab arm (the lower triangle in Fig. 2F) but not in the docetaxel arm (the upper triangle in Fig. 2F).

### Metastatic-organ landscape as a predictor in a PD-L1 dependent manner

**Fig. 3:**
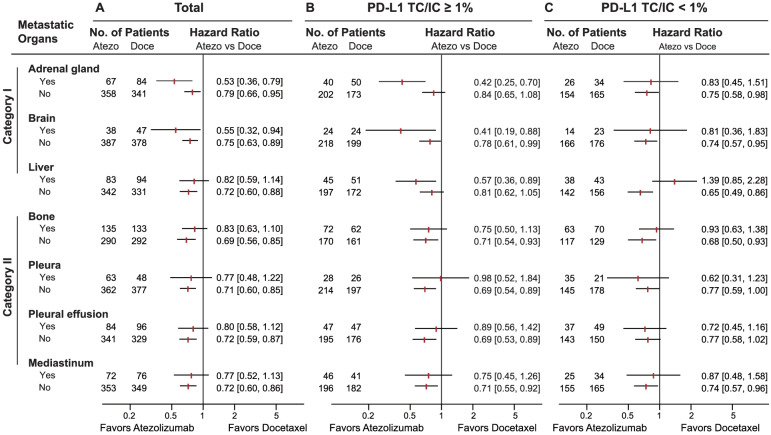
Predictive significance of metastatic-organ landscape for efficacy with immunotherapy versus chemotherapy in a PD-L1-dependent fashion. Forest plots showing overall survival (OS) benefits of atezolizumab versus docetaxel per organ metastatic category in **A** the total, **B** PD-L1-positive (TC/IC ≥ 1%), and **C** PD-L1-negative (TC/IC < 1%) populations from OAK trial. Two categories of metastatic organs were identified based on OS benefits of atezolizumab versus docetaxel in the PD-L1-positive population, and metastases to category I organ (adrenal glands, brain, and liver) presented significantly better OS results in atezolizumab arm versus docetaxel arm, while metastases to category II organ (bone, pleura, pleural effusion, and mediastinum) derived no benefit from atezolizumab over docetaxel. Abbreviations: Atezo, atezolizumab; Doce, docetaxel; CI, confidence interval; PD-L1, programmed death-ligand 1; TC, tumor cell; IC, immune cell

Upon comparing the efficacy following treatment with immunotherapy and chemotherapy, we found an identifiable association between the metastatic-organ landscape and the clinical benefits of atezolizumab versus docetaxel in the total population from OAK (Fig. 3A). On account of the clinical practice of immunotherapy in patients who were PD-L1 positive (≥ 1%), we investigated the predictive effect (immunotherapy versus chemotherapy) of the metastatic-organ landscape stratified by PD-L1 status. Intriguingly, the predictive effect was observed exclusively in the PD-L1-positive population (TC/IC ≥ 1%; Fig. 3B) rather than in the PD-L1-negative population (TC/IC < 1%; Fig. 3C).

In general, the predictive significance varied across metastatic organs at different degrees or even conversely within the PD-L1-positive population, which defined two organ categories (Fig. 3). For category I organs, OS benefits of atezolizumab versus docetaxel were found in patients whose tumors metastasized to adrenal glands (HR 0.42, 95% CI 0.25–0.70), brain (HR 0.41, 95% CI 0.19–0.88), and liver (HR 0.57, 95% CI 0.36–0.89) (Fig. 3B). On the contrary, for bone, pleural, pleural effusion, and mediastinum, patients harboring metastasis to any of these category II organs did not benefit from atezolizumab relative to docetaxel (Fig. 3B). Synergistic predictive effect was found among the category I organ metastases (adrenal glands, brain, and liver) within the PD-L1-positive population (Additional file 4: Fig. S1A-F), where the OS benefits of atezolizumab versus docetaxel in patients with metastases to double category I organs (HR 0.32, 95% CI 0.14-0.76, *P* = 0.0013; Additional file 4: Fig. S1C) were even more pronounced in comparison to those with metastasis to a single category I organ (HR 0.59, 95% CI 0.41-0.85, *P* = 0.0048; Additional file 4: Fig. S1B).

We also examined the predictive versus prognostic effect of the metastatic statuses of category I organs (Additional file 5: Fig. S2A-D). Remarkably within the PD-L1-positvie population, the presence of the category I organ metastases was associated with decreased OS in the docetaxel arm (*P* < 0.0001; Additional file 5: Fig. S2B), indicative of the inherent unfavorable prognostic effect of metastases. In contrast, OS was not influenced by the metastatic number of the category I organs in the atezolizumab arm (*P* = 0.5978; Additional file 5: Fig. S2A), suggesting that the unfavorable prognostic effect was alleviated by the favorable predictive effect specific to immunotherapy. Conversely, the inherent unfavorable prognostic effect of metastases became discernible in PD-L1-negative patients treated with atezolizumab, since the immunotherapeutic predictive effect was absent in this population; consequently, we observed a negative correlation between OS and the number of the category I organ metastases (*P* = 0.0005; Additional file 5: Fig. S2C).

### Incorporating metastatic-organ landscape to forecast ICI therapy

The synergistic value of different metastatic organs inspired us to incorporate the whole landscape for comprehensive assessment. Having identified metastatic-organ landscape as a determinant of both inherent prognosis and immunotherapeutic benefit, we believed that only through incorporating both the treatment-independent prognostic effect and the immunotherapy-specific predictive effect of the whole metastatic-organ landscape could we truly reflect its impact on ICI therapy.

**Fig. 4:**
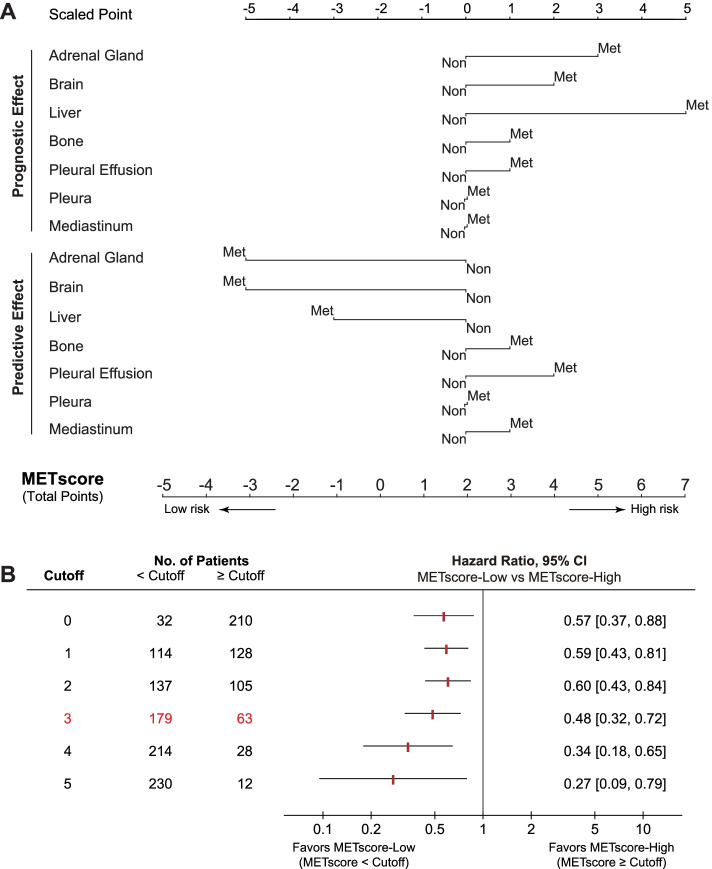
A metastasis-based scoring system (METscore) incorporating prognostic and predictive effects for ICI prediction in the PD-L1-positive population. **A** Nomogram illustrating scaled points as proxy for prognostic and predictive effects of metastatic organs, and the total points as METscore for the prediction of overall survival of PD-L1-positvive patients following ICI therapy. **B** Putative cut-points (0 ~ 5) of METscore to stratify PD-L1-positive patients into -High and -Low groups with significantly different OS in the atezolizumab-treated population from OAK. Abbreviations: Met, metastasis; Non, non-metastasis; CI, confidence interval; OS, overall survival; ICI, immune checkpoint inhibitor; PD-L1, programmed cell death-ligand 1

To this end, we proposed a metastasis-based scoring system (METscore) to forecast survival outcomes of advanced-stage NSCLC patients treated with ICI agents using the PD-L1-positive OAK cohort, since the therapeutic impact was particularly witnessed in this stratum (Fig. 4A). On this basis, given a metastatic profile of a certain patient obtained from pretreatment radiologic assessments, two scaled points could be assigned to each metastatic organ, one of which delineated the prognostic effect and the other delineated the predictive effect. The total points of prognostic and predictive effects of the whole metastatic-organ landscape constituted the METscore in forecasting the survival benefits following ICI therapy.

A range of putative cut-points (0 ~ 5) for METscore was assessed in the atezolizumab population (Fig. 4B). There was a general pattern of prolonged OS in patients with lower METscore relative to those with higher METscore, and an enlarged OS difference could be expected if more stringent cut-points (4 and 5) were selected (Fig. 4B). For practical application, the optimal cut-point of METscore, corresponding to 3 achieved by the maximally selected rank statistics, was determined by weighing the survival benefits against the minimal proportion of each group. And patients were classified into METscore-High (METscore ≥ 3) and -Low (METscore < 3) categories accordingly. Thereupon, the METscore system and the threshold were eventually locked for performance evaluation throughout the study. The system has been translated into a web-based tool that is freely available to the public (Additional file 6: Fig. S3).

### METscore enables identification of therapeutic benefit from checkpoint blockade

**Fig. 5:**
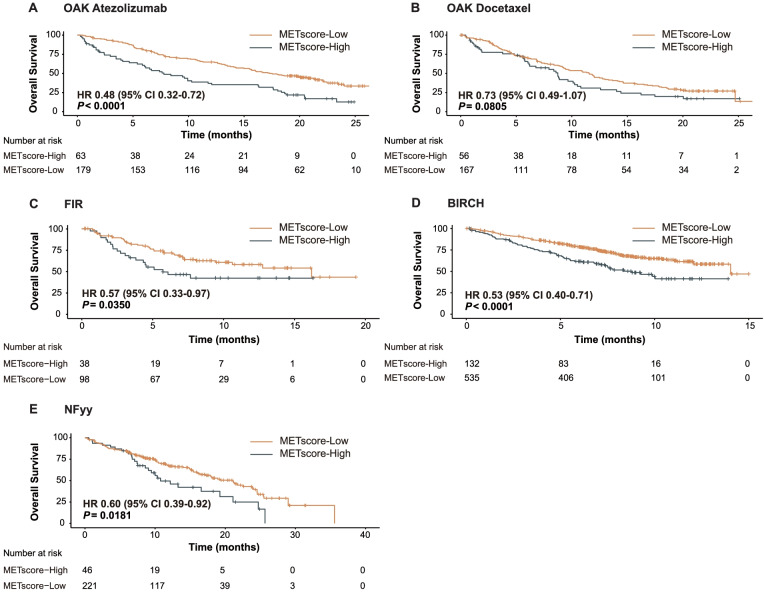
METscore forecasts survival outcomes of checkpoint blockade-based immunotherapy in the PD-L1-positive population. Kaplan-Meier curves showing overall survival according to METscore groups (≥ 3 vs. < 3) in **A** atezolizumab- and **B** docetaxel-treated populations from PD-L1-positive OAK cohort. Kaplan-Meier curves showing overall survival according to METscore groups in PD-L1-selected atezolizumab trials, **C** FIR and **D** BIRCH, and **E** NFyy cohort. Abbreviations: HR, hazard ratio; CI, confidence interval; PD-L1, programmed death-ligand 1

We first evaluated the discrimination performance of METscore in the PD-L1-positive OAK cohort. Patients with METscore-Low obtained significantly longer OS than METscore-High counterparts in the atezolizumab-treated population (HR 0.48, 95% CI 0.32–0.72, *P* < 0.0001; Fig. 5A). By contrast, there was no significant difference in terms of OS between METscore-Low and -High groups in the docetaxel-treated population (HR 0.73, 95% CI 0.49–1.07, *P* = 0.0805; Fig. 5B). Concurrently, upon direct comparison of survival prospects following treatment with immunotherapy and chemotherapy, OS was demonstrated to favor atezolizumab as compared to docetaxel within the METscore-Low group (HR 0.64, 95% CI 0.49–0.84, *P* = 0.0011; Additional file 7: Fig. S4A), whereas it was generally similar between the two arms within the METscore-High group (HR 0.98, 95% CI 0.64–1.48, *P* = 0.9121; Additional file 7: Fig. S4B). These results indicated that METscore enabled noninvasive identification of beneficiaries of ICI therapy, i.e., the METscore-Low patients.

### Generalization of METscore in external clinical-trial and real-world cohorts

The METscore-based system was then externally validated in PD-L1-selected clinical-trial and real-world cohorts. In FIR, the use of METscore allowed stratification of patients into METscore-High and -Low groups with significant difference of OS (HR 0.57, 95% CI 0.33–0.97, *P* = 0.0350; Fig. 5C). In BIRCH, OS rates consistently favored METscore-Low over METscore-High (HR 0.53, 95% CI 0.40–0.71, *P* < 0.0001; Fig. 5). On top of that, the generalization performance of METscore was confirmed in NFyy, where patients with METscore-Low had prolonged OS than those with METscore-High (HR 0.60, 95% CI 0.39–0.92, *P* = 0.0181; Fig. 5E).

**Fig. 6:**
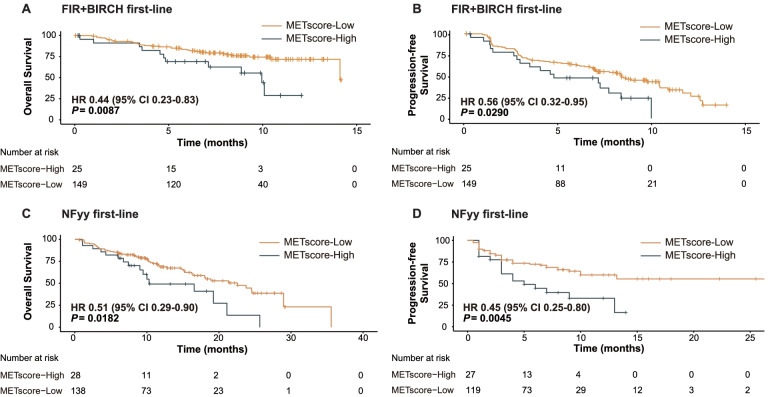
METscore enables classification of survival benefits from first-line checkpoint blockade-based immunotherapy. Kaplan-Meier curves of **A** overall survival and **B** progression-free survival according to METscore groups (≥ 3 vs. < 3) for patients receiving atezolizumab as first-line therapy within PD-L1-selected FIR and BIRCH trials. Kaplan-Meier curves of **C** overall survival and **D** progression-free survival according to METscore groups for patients receiving ICIs as first-line therapy within the PD-L1-selected NFyy cohort. Abbreviations: ICI, immune checkpoint inhibitor; HR, hazard ratio; CI, confidence interval; PD-L1, programmed death-ligand 1

Considering that ICI has become the first-line treatment of advanced NSCLC [17], we evaluated the discrimination performance of METscore specifically in the first-line setting. It is noteworthy that survival prospects were significantly longer for METscore-Low patients referenced to METscore-High patients in the first-line FIR/BIRCH cohort in terms of both OS (HR 0.44, 95% CI 0.23–0.83, *P* = 0.0087; Fig. 6A) and PFS (HR 0.56, 95% CI 0.32–0.95, *P* = 0.0290; Fig. 6B). The significant survival advantage of METscore-Low over METscore-High groups were replicated in the first-line NFyy cohort according to OS (HR 0.51, 95% CI 0.29–0.90, *P* = 0.0182; Fig. 6C) and PFS (HR 0.45, 95% CI 0.25–0.80, *P* = 0.0045; Fig. 6D).

## Discussion

Understanding organ-specific tumor immune context has been one of the top ten challenges in cancer immunotherapy [4]. However, even in an era of precision medicine where regimens are increasingly informed by multi-omic data, the role of metastatic organs in affecting the efficacy of immunotherapy remains unclear. In this study, we delineated and incorporated both predictive and prognostic effects of metastatic-organ landscape to forecast survival outcomes of advanced NSCLC patients following ICI therapy, particularly in the PD-L1-positive stratum.

While cancers originating from various organs presented different susceptibility to ICIs, immunotherapeutic efficacy could even vary greatly among advanced patients with the same cancer type based on anatomically differences of metastatic tumor lesions [4]. Until recently, however, the metastatic-organ landscape has not yet been incorporated into the current clinical practice of immunotherapy in the metastatic setting, partially due to the paucity of evidence; this provided a direction for researchers to dissect therapeutic heterogeneity and to accelerate clinical progress from the dimension of metastatic organ-specific antitumor immunity. With years of concerted efforts, association between metastatic organs and ICI efficacy continued to be uncovered [5, 6, 18, 19]. Nonetheless, owing to the single-arm nature of most previous studies without comparison treatment arms, whether the given metastatic organ was purely a treatment-independent prognostic factor or a immunotherapy-specific predictive factor remains controversial [7]; consequently, confusions would be generated when making claims of the predictive effect under the circumstance of single-arm ICI cohort.

In principle, a predictive marker should be claimed preferably in a randomized cohort [7]. Taking advantage of the double-arm randomized OAK trial of immunotherapy versus traditional chemotherapy, we were able to make the distinction between predictive and prognostic effects. Herein, we dissected the predictive versus prognostic effect of each metastatic organ. In particular, the metastatic-organ landscape exerted immunotherapeutic impact predominantly on the PD-L1-positive (TC/IC ≥ 1%) population. This makes sense as PD-L1 ≥ 1% was exactly the indication for first-line immunotherapy of advanced NSCLC on the basis of the National Comprehensive Cancer Network (NCCN) guidelines [17]. Within the PD-L1-positive stratum, metastatic organs were furtherly divided into two categories according to their survival benefits of immunotherapy over chemotherapy, and the predictive and prognostic effects were delineated utilizing a multivariate Cox regression with the inclusion of treatment-by-organ interaction terms.

Of note, the presence of metastasis to category I organs, including adrenal glands, brain, and liver, was identified as a potential predictive marker of sensitivity to immunotherapy, in spite of the coexisting adverse prognostic effect inherently coupled with this malignant biology behavior of tumors (i.e., metastasis). The paradoxical coexistence of the favorable predictive effect and the unfavorable prognostic effect of category I organ metastases (Fig. 4A) helped explain the non-significant association of survival prospects with the number of category I organ metastases in the immunotherapy arm (Additional file 5: Fig. S2A), which was congruent with accumulating evidence supporting that adrenal gland or brain metastasis did not influence patients’ survival in the context of PD-(L)1 blockade of NSCLC [19, 20]. This was reminiscent of our recent study reporting an upregulated blood-based tumor mutation burden and/or PD-L1 expression for theses metastases [20], and another study reporting a favorable response rate of adrenal gland metastatic lesions to ICIs [18]. The “Janus-faced” significance of category I organ metastases has also been recapitulated in liver metastasis, where superior efficacy of immunotherapy over chemotherapy was coupled with a worse prognosis [21–23]. Interestingly, the presence of liver metastases in PD-L1-negative patients predicts lack of response to immunotherapy (Fig. 3C). Results from two IMvigor211 (NCT02302807) [24, 25] and IMvigor210 (NCT02108652) [26–28] support the finding in a cross-cancer manner, implying that these patients may avoid immunotherapy (Additional file 8: Fig. S5). All these convergence of evidences suggested PD-(L)1 blockade shaped antitumor immunity in a metastatic-organ specific manner.

For first-line therapy of advanced NSCLC without actionable molecular biomarkers, patients tested as PD-L1 positive are recommended to receive immunotherapy according to the NCCN guidelines. Despite that, even in this PD-L1-selected population, the response rate to immunotherapy is mostly less than 50% in first-line trials [29], which prompted us to identify more accurate beneficiaries from immunotherapy through the integration of the organ-specific metastatic landscape. Accordingly, a metastasis-based scoring system was developed to classify PD-L1-positive patients into METscore-High and -Low categories with differential survival benefits derived from ICI agents, which was later on validated in real-life and first-line cohorts. Our results indicate that the METscore system might provide further guidance for therapeutic strategies of advanced NSCLC patients in the context of PD-L1 positive. In view of the efficacy benefits over chemotherapy in METscore-Low patients, immunotherapy might be a preferable alternative for these patients. As for METscore-High patients that account for a relatively small proportion, it is rational to treat them with conventional therapy due to a lack of response to immunotherapy, which could also avoid the risk of developing immune-related toxicity and hyperprogression.

As survival gains have been witnessed in the addition of bevacizumab to immunochemotherapy among first-line populations, including those difficult-to-treated patients harboring liver metastases, this combination strategy is approved for the treatment of metastatic nonsquamous NSCLC without EGFR/ALK genetic alterations [1, 30, 31]. To evaluate the discrimination performance of METscore in this regimen, we applied the system to patients receiving ICIs plus bevacizumab and chemotherapy in the NFyy cohort. We found that OS trended longer in the METscore-Low group compared to its counterpart; concordantly, both OS and PFS rates favored METscore-Low over METscore-High among patients in the first-line setting, albeit not reaching a conventional significant level (Additional file 9: Fig. S6). These findings indicate the METscore system might also play its role in patients receiving bevacizumab in combination with immunochemotherapy. Nonetheless, the findings should be interpreted with caution due to the small sample size; a more holistic assessment in cohorts with immunotherapy-based combination regimens is needed before its reliable utility in the complicated clinical practice.

Moreover, we noted that the effect of the category I metastatic organs in the PD-L1-negative population was the opposite of that in the PD-L1 positive population (Additional file 5: Fig. S2). OS was generally similar regardless of the category I metastases in PD-L1-negative patients treated with docetaxel (*P* = 0.5766; Additional file 5: Fig. S2D), which is in line with the results illustrated in Fig. 3C. In the PD-L1-negative stratum, patients without metastases to adrenal glands, brain, or liver derived significant clinical benefits from atezolizumab over docetaxel, whereas the superiority was removed or even reversed in patients with these metastases, particularly liver metastasis, resulting in an absence of significant difference. We also evaluated METscore in the PD-L1-negative stratum in OAK without external validation and observed moderate discrimination performance for risk stratification in both arms (Additional file 10: Fig. S7). Since METscore is the sum of scaled points from both predictive effect and prognostic effect (Methods, Fig. 4A), we reasoned that it is the prognostic component of METscore that contribute to its moderate ability in the PD-L1-negative population where the immunotherapeutic predictive component does not work. Therefore, its performance in patients tested as PD-L1 negative or treated with non-ICI therapies cannot be guaranteed.

Limitations existed in this study. Firstly, lung metastasis was not taken into account, since it was not identified as ipsilateral or contralateral in the trial data while our study focused on distant metastases. Secondly, METscore has its own scope of application, namely in the PD-L1-positive population treated with immunotherapy. Additionally, the study was based on post hoc analyses; despite the METscore model was independently validated using clinical-trial and real-word cohort as well as the first-line subsets, prospective validation of the METscore model is still needed in a clinical trial setting to corroborate these findings.

## Conclusions

In conclusion, the study delineated the immunotherapeutic predictive versus prognostic effect of metastatic-organ landscape for advanced NSCLC patients in a PD-L1-dependent manner, which helped to improve the understanding of organ-specific antitumor immunity in cancer immunotherapy. Using baseline radiologic assessments, our metastasis-based scoring system incorporating both predictive and prognostic effects of metastatic-organ landscape enabled non-invasive forecast of survival outcomes in PD-L1-positive NSCLC patients following ICI therapy.

## Supplementary Information

**Additional file 1: Table S1.** Clinical characteristics of the NFyy cohort.

**Additional file 2: Table S2.** The prognostic effect (regardless of treatments) and the predictive effect (immunotherapy versus chemotherapy) of each metastatic organ for overall survival within the PD-L1-positive OAK cohort delineated via multivariable Cox proportional hazard model.

**Additional file 3: Table S3.** Metastasis-based risk schema to measure survival outcomes for the PD-L1-positive population following immune checkpoint therapy.

**Additional file 4: Figure S1.** Efficacy of atezolizumab versus docetaxel according to category I organ metastases (adrenal glands, brain, and liver) and PD-L1 status. Kaplan-Meier curves showing overall survival benefits of atezolizumab versus docetaxel in patients whose tumors were metastasized to (A) none, (B) any, and (C) two of the category I organs in the PD-L1-positive (TC/IC ≥ 1%) population. Kaplan-Meier curves showing overall survival benefits of atezolizumab versus docetaxel in patients whose tumors were metastasized to (D) none, (E) any, and (F) two of the category I organs in the PD-L1-negative (TC/IC < 1%) population. Abbreviations: Met, metastasis; HR, hazard ratio; CI, confidence interval; PD-L1, programmed cell death-ligand 1; TC, tumor cell; IC, immune cell.

**Additional file 5: Figure S2.** Category I organ metastases (adrenal glands, brain, and liver) for survival outcomes of atezolizumab- and docetaxel-treated populations stratified by PD-L1 status. Kaplan-Meier curves showing overall survival according to category I organ metastases in (A) atezolizumab- and (B) docetaxel- treated patients with PD-L1 TC/IC ≥ 1%. Kaplan-Meier curves showing overall survival according to category I organ metastases in (C) atezolizumab- and (D) docetaxel- treated patients with PD-L1 TC/IC < 1%. Abbreviations: Met, metastasis; PD-L1, programmed cell death-ligand 1; TC, tumor cell; IC, immune cell.

**Additional file 6: Figure S3.** A web-based tool for METscore calculation. (A) An illustration of the use of the web server (https://metscore-ici.github.io/Pages/) for calculating METscore for ICI-treated NSCLC patients and (B) Four examples. Abbreviations: ICI, immune checkpoint inhibitor; NSCLC, non-small cell lung cancer.

**Additional file 7: Figure S4.** Efficacy of atezolizumab versus docetaxel stratified by METscore in PD-L1-positive OAK cohort. Kaplan-Meier curves showing overall survival benefits of atezolizumab versus docetaxel in (A) METscore-Low and (B) METscore-High populations with PD-L1 TC/IC ≥ 1% from OAK trial. Abbreviations: HR, hazard ratio; CI, confidence interval; PD-L1, programmed cell death-ligand 1; TC, tumor cell; IC, immune cell.

**Additional file 8: Figure S5.** Atezolizumab efficacy in PD-L1-negative patients with liver metastases. (A) Kaplan-Meier curve of overall survival comparing atezolizumab and chemotherapy in PD-L1-negative patients with liver metastases in IMvigor211. (B) Kaplan-Meier curve of overall survival in atezolizumab-treated patients with liver metastases stratified by PD-L1 status in IMvigor211. Abbreviations: HR, hazard ratio; CI, confidence interval; PD-L1, programmed death-ligand 1.

**Additional file 9: Figure S6.** Discrimination performance of METscore for combined immunochemotherapy and bevacizumab in the NFyy cohort. (A) Kaplan-Meier curve of overall survival according to METscore groups (≥ 3 vs. < 3) in patients receiving ICIs plus bevacizumab and chemotherapy. Kaplan-Meier curves of (B) overall survival and (C) progression-free survival according to METscore groups in patients receiving ICIs plus bevacizumab and chemotherapy as first-line treatment. Abbreviations: ICI, immune checkpoint inhibitor; HR, hazard ratio; CI, confidence interval; PD-(L)1, programmed death-(ligand) 1.

**Additional file 10: Figure S7.** Forest plots showing overall survival benefits of METscore-Low over METscore-High per PD-L1 status in (A) docetaxel and (B) atezolizumab arms in OAK. Abbreviations: HR, hazard ratio; CI, confidence interval; PD-L1, programmed death-ligand 1.

## Data Availability

A web-based tool for calculating METscore is available at https://metscore-ici.github.io/Pages/. Data are available upon reasonable request from corresponding authors, Zhong-Yi Dong (dongzy1317@foxmail.com) and Jian-Guo Zhou (jianguo.zhou@yahoo.com).

## Abbreviations

ICI: Immune checkpoint inhibitor
NSCLC: Non-small cell lung cancer
PD-1: Programmed death-1
PD-L1: Programmed death-ligand 1
TC: Tumor cell
IC: Immune cell
TPS: Tumor proportion score
OS: Overall survival
PFS: Progression-free survival
HR: Hazard ratio
CI: Confidence interval

## References

[1] Socinski MA, Jotte RM, Cappuzzo F, Orlandi F, Stroyakovskiy D, Nogami N (2018). Atezolizumab for first-line treatment of metastatic nonsquamous NSCLC. N Engl J Med.

[2] Zou W, Wolchok JD, Chen L (2016). PD-L1 (B7-H1) and PD-1 pathway blockade for cancer therapy: mechanisms, response biomarkers, and combinations. Sci Transl Med.

[3] Pao W, Ooi CH, Birzele F, Ruefli-Brasse A, Cannarile MA, Reis B (2018). Tissue-specific immunoregulation: a call for better understanding of the “immunostat” in the context of cancer. Cancer Discov.

[4] Hegde PS, Chen DS (2020). Top 10 challenges in cancer immunotherapy. Immunity..

[5] Tamiya M, Tamiya A, Inoue T, Kimura M, Kunimasa K, Nakahama K (2018). Metastatic site as a predictor of nivolumab efficacy in patients with advanced non-small cell lung cancer: a retrospective multicenter trial. PLoS One.

[6] Huang Y, Zhu L, Guo T, Chen W, Zhang Z, Li W (2021). Metastatic sites as predictors in advanced NSCLC treated with PD-1 inhibitors: a systematic review and meta-analysis. Hum Vaccin Immunother.

[7] Ballman KV (2015). Biomarker: predictive or prognostic?. J Clin Oncol.

[8] A Phase III, Open-Label, Multicenter, Randomized study to investigate the efficacy and safety of atezolizumab (anti-PD-L1 antibody) compared with docetaxel in patients with non-small cell lung cancer after failure with platinum containing chemotherapy (OAK). Vivli 10.25934/00005794 (2017).

[9] A Phase II, Multicenter, single-arm study of MPDL3280A in patients with PD-L1-positive locally advanced or metastatic non-small cell lung cancer. Vivli 10.25934/00005791 (2018).

[10] A Phase II, Multicenter, single-arm study of atezolizumab in patients with PD-L1-positive locally advanced or metastatic non-small cell lung cancer (BIRCH). Vivli 10.25934/00005796 (2017).

[11] Rittmeyer A, Barlesi F, Waterkamp D, Park K, Ciardiello F, von Pawel J (2017). Atezolizumab versus docetaxel in patients with previously treated non-small-cell lung cancer (OAK): a phase 3, open-label, multicentre randomised controlled trial. Lancet..

[12] Gandara DR, Paul SM, Kowanetz M, Schleifman E, Zou W, Li Y (2018). Blood-based tumor mutational burden as a predictor of clinical benefit in non-small-cell lung cancer patients treated with atezolizumab. Nat Med.

[13] Spigel DR, Chaft JE, Gettinger S, Chao BH, Dirix L, Schmid P (2018). FIR: efficacy, safety, and biomarker analysis of a phase II open-label study of atezolizumab in PD-L1-selected patients with NSCLC. J Thorac Oncol.

[14] Peters S, Gettinger S, Johnson ML, Jänne PA, Garassino MC, Christoph D (2017). Phase II trial of atezolizumab as first-line or subsequent therapy for patients with programmed death-ligand 1-selected advanced non-small-cell lung cancer (BIRCH). J Clin Oncol.

[15] Hopkins AM, Kichenadasse G, Garrett-Mayer E, Karapetis CS, Rowland A, Sorich MJ (2020). Development and validation of a prognostic model for patients with advanced lung cancer treated with the immune checkpoint inhibitor atezolizumab. Clin Cancer Res.

[16] Hurria A, Togawa K, Mohile SG, Owusu C, Klepin HD, Gross CP (2011). Predicting chemotherapy toxicity in older adults with cancer: a prospective multicenter study. J Clin Oncol.

[17] Ettinger DS, Wood DE, Aggarwal C, Aisner DL, Akerley W, Bauman JR (2019). NCCN guidelines insights: non-small cell lung cancer, version 1.2020. J Natl Compr Cancer Netw.

[18] Osorio JC, Arbour KC, Le DT, Durham JN, Plodkowski AJ, Halpenny DF (2019). Lesion-level response dynamics to programmed cell death protein (PD-1) blockade. J Clin Oncol.

[19] Zhang G, Cheng R, Wang H, Zhang Y, Yan X, Li P (2020). Comparable outcomes of nivolumab in patients with advanced NSCLC presenting with or without brain metastases: a retrospective cohort study. Cancer Immunol Immunother.

[20] Ma SC, Tang XR, Long LL, Bai X, Zhou JG, Duan ZJ (2021). Integrative evaluation of primary and metastatic lesion spectrum to guide anti-PD-L1 therapy of non-small cell lung cancer: results from two randomized studies. Oncoimmunology..

[21] Qin BD, Jiao XD, Liu J, Liu K, He X, Wu Y (2020). The effect of liver metastasis on efficacy of immunotherapy plus chemotherapy in advanced lung cancer. Crit Rev Oncol Hematol.

[22] Li S, Sun S, Xiang H, Yang J, Peng M, Gao Q (2020). Liver metastases and the efficacy of the PD-1 or PD-L1 inhibitors in cancer: a meta-analysis of randomized controlled trials. Oncoimmunology..

[23] Yin WJ, Ma SC, Dong ZY, Xu M, Mao W (2021). Efficacy and treatment strategies in advanced cancers with liver metastasis receiving atezolizumab therapy. Cancer Manag Res.

[24] van der Heijden MS, Loriot Y, Durán I, Ravaud A, Retz M, Vogelzang NJ (2021). Atezolizumab versus chemotherapy in patients with platinum-treated locally advanced or metastatic urothelial carcinoma: a long-term overall survival and safety update from the phase 3 IMvigor211 clinical trial. Eur Urol.

[25] A phase III, open-label, multicenter, randomized study to investigate the efficacy and safety of atezolizumab (anti-PD-L1 antibody) Compared with chemotherapy in patients with locally advanced or metastatic urothelial bladder cancer after failure with platinum-containing chemotherapy. Vivli 10.25934/00005598 (2018).

[26] Balar AV, Galsky MD, Rosenberg JE, Powles T, Petrylak DP, Bellmunt J (2017). Atezolizumab as first-line treatment in cisplatin-ineligible patients with locally advanced and metastatic urothelial carcinoma: a single-arm, multicentre, phase 2 trial. Lancet..

[27] Mariathasan S, Turley SJ, Nickles D, Castiglioni A, Yuen K, Wang Y (2018). TGFβ attenuates tumour response to PD-L1 blockade by contributing to exclusion of T cells. Nature..

[28] A phase II, multicenter, single-arm study of atezolizumab in patients with locally advanced or metastatic urothelial bladder cancer. Vivli. 10.25934/00005804 (2017).

[29] Morad G, Helmink BA, Sharma P, Wargo JA (2021). Hallmarks of response, resistance, and toxicity to immune checkpoint blockade. Cell..

[30] Nogami N, Barlesi F, Socinski MA, Reck M, Thomas CA, Cappuzzo F, et al. IMpower150 final exploratory analyses for atezolizumab plus bevacizumab and chemotherapy in key NSCLC patient subgroups with EGFR mutations or metastases in the liver or brain. J Thorac Oncol. 2021. 10.1016/j.jtho.2021.09.014. 10.1016/j.jtho.2021.09.014 34626838

[31] Reck M, Mok TSK, Nishio M, Jotte RM, Cappuzzo F, Orlandi F (2019). Atezolizumab plus bevacizumab and chemotherapy in non-small-cell lung cancer (IMpower150): key subgroup analyses of patients with EGFR mutations or baseline liver metastases in a randomised, open-label phase 3 trial. Lancet Respir Med.

