# Supplementary material for: Organ-specific metastatic landscape dissects PD-(L)1 blockade efficacy in advanced non-small cell lung cancer: applicability from clinical trials to real-world practice
Source: BMC Med. 2022 Apr 12;20:120. doi: 10.1186/s12916-022-02315-2 (PMC9004108; doi:10.1186/s12916-022-02315-2)
Supplement: Supplementary file 1 — Additional file 1: Table S1. Clinical characteristics of the NFyy cohort. [file 12916_2022_2315_MOESM1_ESM.docx]

**Additional file 1: Table S1.** Clinical characteristics of the NFyy cohort.

| **Clinical characteristics** | **n (%) / mean (SD)** |
| --- | --- |
| **Stage** |  |
| IV | 409 (100) |
| **Gender** |  |
| Male (%) | 305 (74.6) |
| Female (%) | 104 (25.4) |
| **Age** [mean (SD)] | 58.43 (10.80) |
| **Body mass index** [mean (SD)] | 22.11 (3.25) |
| **Histological type** |  |
| Adenocarcinoma (%) | 261 (63.8) |
| Squamous cell carcinoma (%) | 109 (26.7) |
| Large cell lung cancer (%) | 7 (1.7) |
| Others (%) | 16 (3.9) |
| Not available (%) | 16 (3.9) |
| **Smoking status** |  |
| Never (%) | 178 (43.5) |
| Ever/present (%) | 192 (46.9) |
| Not available (%) | 39 (9.5) |
| **Pack year** [mean (SD)] | 24.54 (36.50) |
| **Treatment line** |  |
| first line (%) | 203 (49.6) |
| Subsequent lines (%) | 133 (32.5) |
| Not available (%) | 73 (17.8) |
| **Treatment strategy** |  |
| Monotherapy (%) | 102 (24.9) |
| Combination therapy (%) | 245 (59.9) |
| Not available (%) | 62 (15.2) |
| **Metastatic organs** |  |
| Adrenal gland (%) | 68 (16.7) |
| Bone (%) | 203 (49.6) |
| Brain (%) | 104 (25.4) |
| Liver (%) | 82 (20.0) |
| Mediastinum (%) | 42 (10.3) |
| Pleura (%) | 146 (35.7) |
| Pleural effusion (%) | 41 (10.0) |
| **EGFR status** |  |
| Positive (%) | 71 (17.4) |
| Negative (%) | 332 (81.2) |
| Not available (%) | 6 (1.4) |
| **ALK status** |  |
| Positive (%) | 10 (2.4) |
| Negative (%) | 224 (54.8) |
| Not available (%) | 175 (42.8) |
| **MET status** |  |
| 1+ (%) | 39 (9.5) |
| 2+ (%) | 41 (10.0) |
| 3+ (%) | 35 (8.6) |
| Negative (%) | 23 (5.6) |
| Not available (%) | 271 (66.3) |
| **HER2 status** |  |
| 1+ (%) | 44 (10.8) |
| 2+ (%) | 16 (3.9) |
| 3+ (%) | 3 (0.7) |
| Negative (%) | 69 (16.9) |
| Not available (%) | 277 (67.7) |
| **BRAF (v600E) status** |  |
| Positive (%) | 5 (1.2) |
| Negative (%) | 146 (35.7) |
| Not available (%) | 258 (63.1) |
| **PD-L1 status** |  |
| Positive (%) | 367 (89.7) |
| Negative (%) | 42 (10.3) |
| **Ki-67 expression** [mean (SD)] | 0.48 (0.30) |

Abbreviation: PD-L1, programmed death-ligand 1; SD, standard deviation.
