# Supplementary material for: Organ-specific metastatic landscape dissects PD-(L)1 blockade efficacy in advanced non-small cell lung cancer: applicability from clinical trials to real-world practice
Source: BMC Med. 2022 Apr 12;20:120. doi: 10.1186/s12916-022-02315-2 (PMC9004108; doi:10.1186/s12916-022-02315-2)
Supplement: Supplementary file 2 — Additional file 2: Table S2. The prognostic effect (regardless of treatments) and the predictive effect (immunotherapy versus chemotherapy) of each metastatic organ for overall survival within the PD-L1-positive OAK cohort delineated via multivariable Cox proportional hazard model. [file 12916_2022_2315_MOESM2_ESM.docx]

# Additional file 2: Table S2. The prognostic effect (regardless of treatments) and the predictive effect (immunotherapy versus chemotherapy) of each metastatic organ for overall survival within the PD-L1-positive OAK cohort delineated via multivariable Cox proportional hazard model.

|  | Variable | Coefficient | HR | 95% CI | Scaled Point^‡^ |
| --- | --- | --- | --- | --- | --- |
| Prognostic Effect* | Adrenal Gland Met | | |  |  |
|  | No | Ref |  |  | **0** |
|  | Yes | 0.6538 | 1.9229 | 1.3056-2.8321 | **3** |
|  | Brain Met | | |  |  |
|  | No | Ref |  |  | **0** |
|  | Yes | 0.3283 | 1.3886 | 0.8151-2.3656 | **2** |
|  | Liver Met | | |  |  |
|  | No | Ref |  |  | **0** |
|  | Yes | 1.0079 | 2.7398 | 1.8895-3.9729 | **5** |
|  | Bone Met | | |  |  |
|  | No | Ref |  |  | **0** |
|  | Yes | 0.2325 | 1.2617 | 0.8808-1.8073 | **1** |
|  | Pleural Effusion Met | | |  |  |
|  | No | Ref |  |  | **0** |
|  | Yes | 0.1479 | 1.1594 | 0.7655-1.7559 | **1** |
|  | Pleural Met | | |  |  |
|  | No | Ref |  |  | **0** |
|  | Yes | 0.0854 | 1.0892 | 0.6373-1.8614 | **0** |
|  | Mediastinum Met | | |  |  |
|  | No | Ref |  |  | **0** |
|  | Yes | 0.0083 | 1.0084 | 0.6553-1.5515 | **0** |
| Predictive Effect^†^ | Adrenal Gland Met | | |  |  |
|  | No | Ref |  |  | **0** |
|  | Yes | -0.9604 | 0.3827 | 0.2115-0.6926 | **-5** |
|  | Brain Met | | |  |  |
|  | No | Ref |  |  | **0** |
|  | Yes | -0.9913 | 0.3711 | 0.1641-0.8394 | **-5** |
|  | Liver Met | | |  |  |
|  | No | Ref |  |  | **0** |
|  | Yes | -0.5276 | 0.5900 | 0.3377-1.0309 | **-3** |
|  | Bone Met | | |  |  |
|  | No | Ref |  |  | **0** |
|  | Yes | 0.1835 | 1.2014 | 0.7217-2.0001 | **1** |
|  | Pleural Effusion Met | | |  |  |
|  | No | Ref |  |  | **0** |
|  | Yes | 0.3981 | 1.4890 | 0.8217-2.6983 | **2** |
|  | Pleural Met | | |  |  |
|  | No | Ref |  |  | **0** |
|  | Yes | 0.0783 | 1.0815 | 0.5166-2.2642 | **0** |
|  | Mediastinum Met | | |  |  |
|  | No | Ref |  |  | **0** |
|  | Yes | 0.1755 | 1.1918 | 0.6653-2.1348 | **1** |

Abbreviations: PD-L1: programmed death-ligand 1; TC: tumor cell; IC: immune cell; HR: hazard ratio; CI: confidence interval; Ref: reference; Met: metastasis. *Prognostic effect depicted the survival benefit of metastasis versus non-metastasis of organs regardless of treatments. ^†^Predictive effect corresponding to treatment-by-organ interaction terms depicted the survival benefit of immunotherapy versus chemotherapy. ^‡^Coefficients of variables were scaled from -5 to 5 and rounded to the nearest whole number to facilitate clinical use.
