# Supplementary material for: Organ-specific metastatic landscape dissects PD-(L)1 blockade efficacy in advanced non-small cell lung cancer: applicability from clinical trials to real-world practice
Source: BMC Med. 2022 Apr 12;20:120. doi: 10.1186/s12916-022-02315-2 (PMC9004108; doi:10.1186/s12916-022-02315-2)
Supplement: Supplementary file 3 — Additional file 3: Table S3. Metastasis-based risk schema to measure survival outcomes for the PD-L1-positive population following immune checkpoint therapy. [file 12916_2022_2315_MOESM3_ESM.docx]

# Additional file 3: Table S3. Metastasis-based risk schema to measure survival outcomes for the PD-L1-positive population following immune checkpoint therapy.

|  |  | -5 | -4 | -3 | -2 | -1 | 0 | 1 | 2 | 3 | 4 | 5 |
| --- | --- | --- | --- | --- | --- | --- | --- | --- | --- | --- | --- | --- |
| Prognostic Effect | Adrenal Gland Met |  |  |  |  |  | N |  |  | Y |  |  |
|  | Brain Met |  |  |  |  |  | N |  | Y |  |  |  |
|  | Liver Met |  |  |  |  |  | N |  |  |  |  | Y |
|  | Bone Met |  |  |  |  |  | N | Y |  |  |  |  |
|  | Pleural Effusion Met |  |  |  |  |  | N | Y |  |  |  |  |
|  | Pleural Met |  |  |  |  |  | N/Y |  |  |  |  |  |
|  | Mediastinum Met |  |  |  |  |  | N/Y |  |  |  |  |  |
| Predictive Effect | Adrenal Gland Met | Y |  |  |  |  | N |  |  |  |  |  |
|  | Brain Met | Y |  |  |  |  | N |  |  |  |  |  |
|  | Liver Met |  |  | Y |  |  | N |  |  |  |  |  |
|  | Bone Met |  |  |  |  |  | N | Y |  |  |  |  |
|  | Pleural Effusion Met |  |  |  |  |  | N |  | Y |  |  |  |
|  | Pleural Met |  |  |  |  |  | N/Y |  |  |  |  |  |
|  | Mediastinum Met |  |  |  |  |  | N | Y |  |  |  |  |

Abbreviations: PD-L1: programmed death-ligand 1; TC: tumor cell; IC: immune cell; Met: metastasis; N: No; Y: Yes.
