# Supplementary figures and images for: Organ-specific metastatic landscape dissects PD-(L)1 blockade efficacy in advanced non-small cell lung cancer: applicability from clinical trials to real-world practice
Source: BMC Med. 2022 Apr 12;20:120. doi: 10.1186/s12916-022-02315-2 (PMC9004108; doi:10.1186/s12916-022-02315-2)

## Category I organs (Adrenal gland / Brain / Liver)

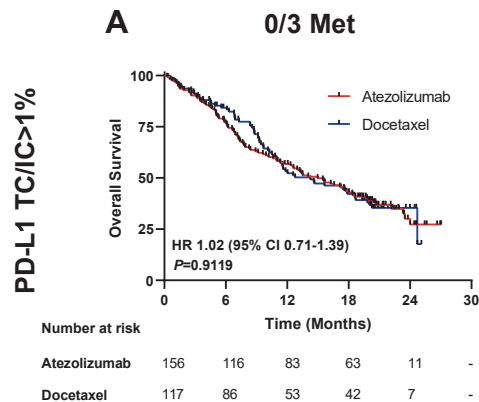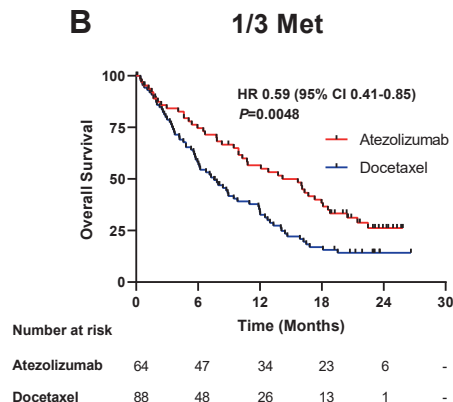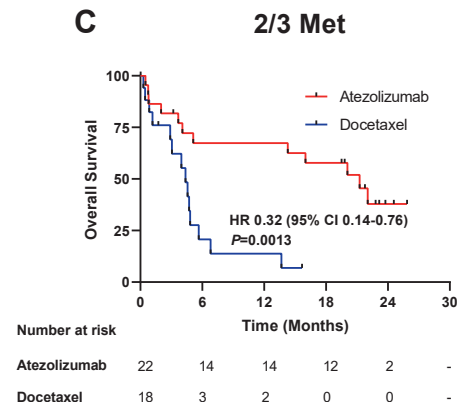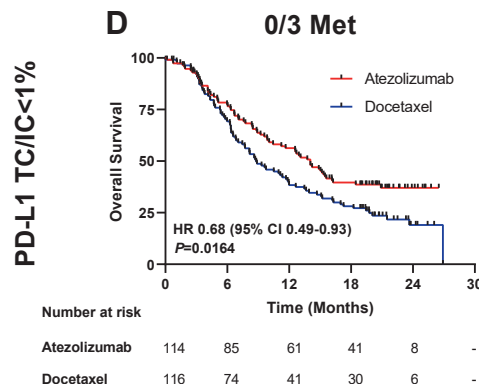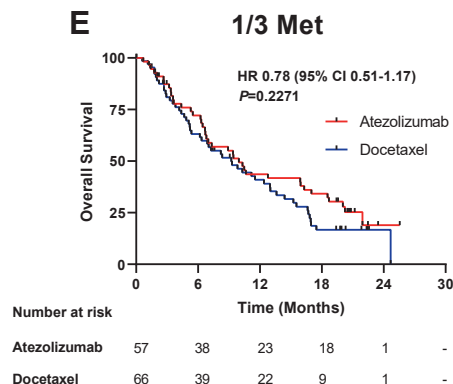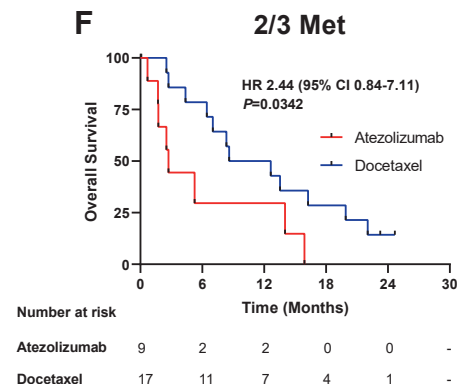

Supplement: Supplementary file 4 — Additional file 4: Figure S1. Efficacy of atezolizumab versus docetaxel according to category I organ metastases (adrenal glands, brain, and liver) and PD-L1 status. Kaplan-Meier curves showing overall survival benefits of atezolizumab versus docetaxel in patients whose tumors were metastasized to (A) none, (B) any, and (C) two of the category I organs in the PD-L1-positive (TC/IC ≥ 1%) population. Kaplan-Meier curves showing overall survival benefits of atezolizumab versus docetaxel in patients whose tumors were metastasized to (D) none, (E) any, and (F) two of the category I organs in the PD-L1-negative (TC/IC < 1%) population. Abbreviations: Met, metastasis; HR, hazard ratio; CI, confidence interval; PD-L1, programmed cell death-ligand 1; TC, tumor cell; IC, immune cell. [file 12916_2022_2315_MOESM4_ESM.pdf]

## A OAK METscore-Low

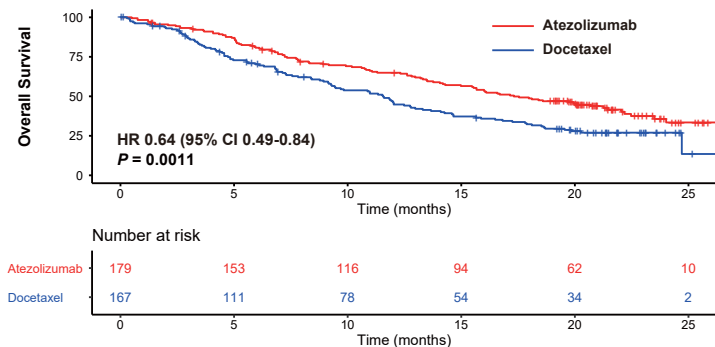

## B OAK METscore-High

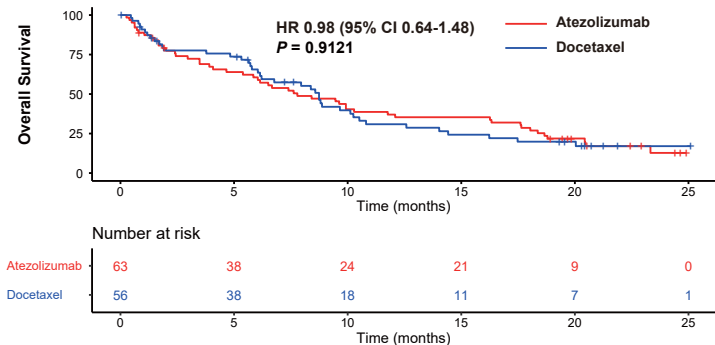

Supplement: Supplementary file 7 — Additional file 7: Figure S4. Efficacy of atezolizumab versus docetaxel stratified by METscore in PD-L1-positive OAK cohort. Kaplan-Meier curves showing overall survival benefits of atezolizumab versus docetaxel in (A) METscore-Low and (B) METscore-High populations with PD-L1 TC/IC ≥ 1% from OAK trial. Abbreviations: HR, hazard ratio; CI, confidence interval; PD-L1, programmed cell death-ligand 1; TC, tumor cell; IC, immune cell. [file 12916_2022_2315_MOESM7_ESM.pdf]

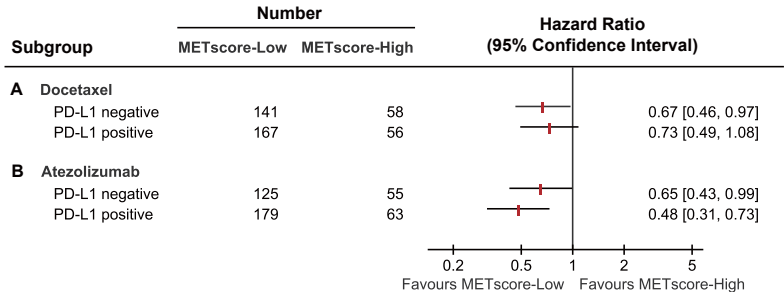

Supplement: Supplementary file 10 — Additional file 10: Figure S7. Forest plots showing overall survival benefits of METscore-Low over METscore-High per PD-L1 status in (A) docetaxel and (B) atezolizumab arms in OAK. Abbreviations: HR, hazard ratio; CI, confidence interval; PD-L1, programmed death-ligand 1. [file 12916_2022_2315_MOESM10_ESM.pdf]
