# Supplementary material for: Organ-specific metastatic landscape dissects PD-(L)1 blockade efficacy in advanced non-small cell lung cancer: applicability from clinical trials to real-world practice
Source: BMC Med. 2022 Apr 12;20:120. doi: 10.1186/s12916-022-02315-2 (PMC9004108; doi:10.1186/s12916-022-02315-2)
Supplement: Supplementary file 5 — Additional file 5: Figure S2. Category I organ metastases (adrenal glands, brain, and liver) for survival outcomes of atezolizumab- and docetaxel-treated populations stratified by PD-L1 status. Kaplan-Meier curves showing overall survival according to category I organ metastases in (A) atezolizumab- and (B) docetaxel- treated patients with PD-L1 TC/IC ≥ 1%. Kaplan-Meier curves showing overall survival according to category I organ metastases in (C) atezolizumab- and (D) docetaxel- treated patients with PD-L1 TC/IC < 1%. Abbreviations: Met, metastasis; PD-L1, programmed cell death-ligand 1; TC, tumor cell; IC, immune cell. [file 12916_2022_2315_MOESM5_ESM.pdf]

Category I organs (Adrenal gland / Brain / Liver)

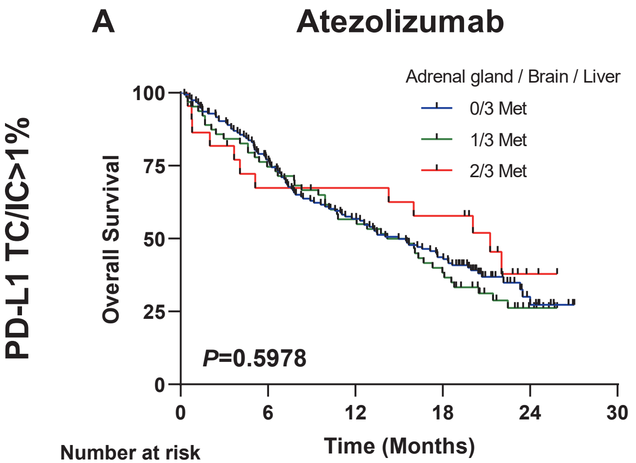

Number at risk

	0	6	12	18	24	30
**0/3 Met**	156	116	83	63	11	-
**1/3 Met**	64	47	34	23	6	-
**2/3 Met**	22	14	14	12	2	-

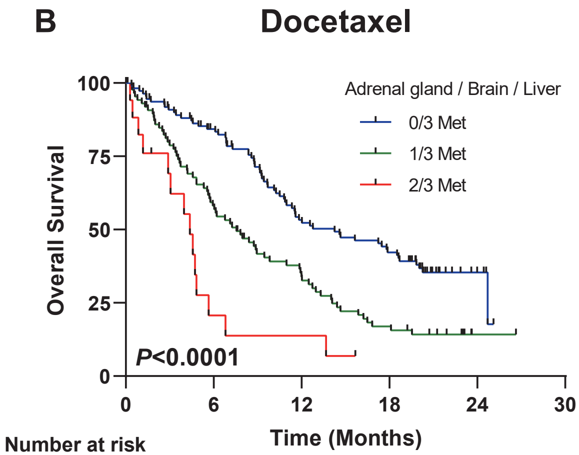

Number at risk

	0	6	12	18	24	30
**0/3 Met**	117	86	53	42	7	-
**1/3 Met**	88	48	26	13	1	-
**2/3 Met**	18	3	2	0	0	-

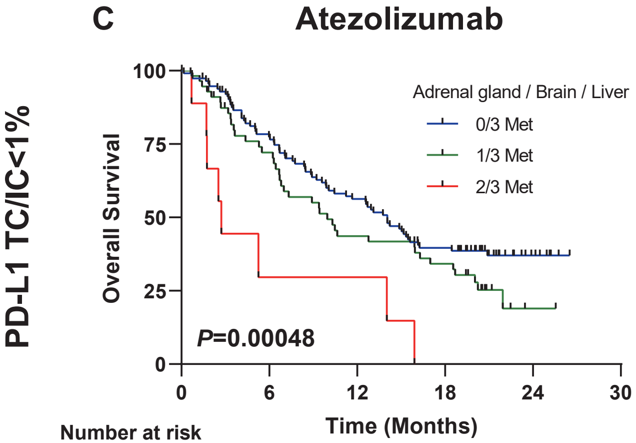

Number at risk

	0	6	12	18	24	30
**0/3 Met**	114	85	61	41	8	-
**1/3 Met**	57	38	23	18	1	-
**2/3 Met**	9	2	2	0	0	-

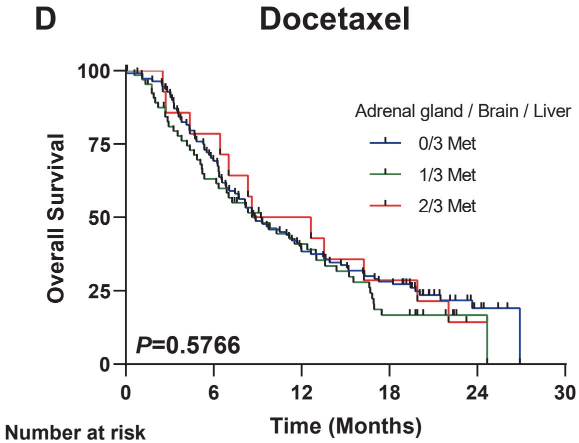

Number at risk

	0	6	12	18	24	30
**0/3 Met**	116	74	41	30	6	-
**1/3 Met**	66	39	22	9	1	-
**2/3 Met**	17	11	7	4	1	-
