# Supplementary material for: Organ-specific metastatic landscape dissects PD-(L)1 blockade efficacy in advanced non-small cell lung cancer: applicability from clinical trials to real-world practice
Source: BMC Med. 2022 Apr 12;20:120. doi: 10.1186/s12916-022-02315-2 (PMC9004108; doi:10.1186/s12916-022-02315-2)
Supplement: Supplementary file 8 — Additional file 8: Figure S5. Atezolizumab efficacy in PD-L1-negative patients with liver metastases. (A) Kaplan-Meier curve of overall survival comparing atezolizumab and chemotherapy in PD-L1-negative patients with liver metastases in IMvigor211. (B) Kaplan-Meier curve of overall survival in atezolizumab-treated patients with liver metastases stratified by PD-L1 status in IMvigor211. Abbreviations: HR, hazard ratio; CI, confidence interval; PD-L1, programmed death-ligand 1. [file 12916_2022_2315_MOESM8_ESM.pdf]

A **IMvigor211 PD-L1 negative patients with liver metastases**

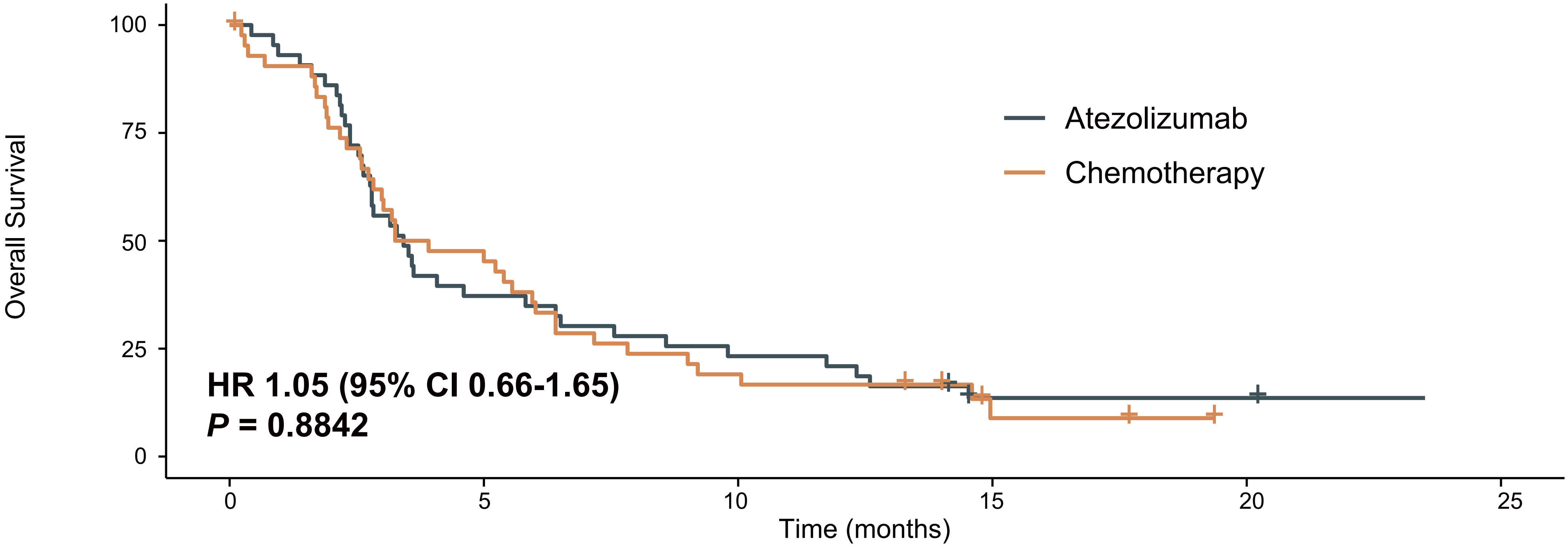

Number at risk

Atezolizumab	43	16	10	4	4	0
Chemotherapy	43	19	8	2	0	0

B **IMvigor210 patients with liver metastases**

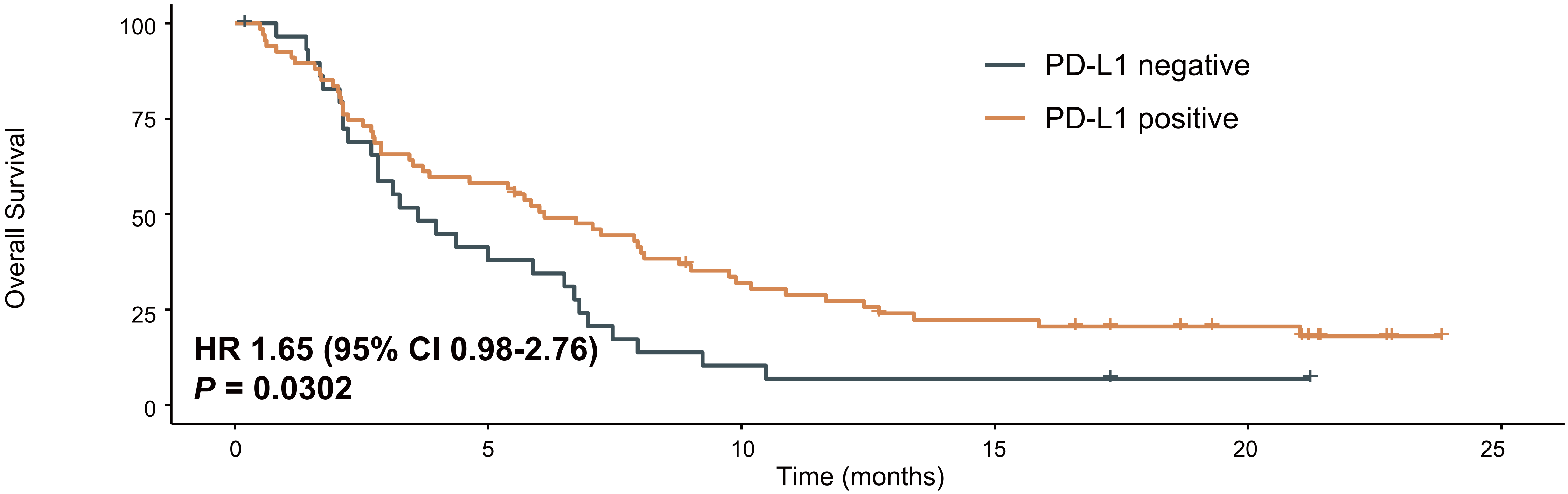

Number at risk

PD-L1 negative	30	11	3	2	1	0
PD-L1 positive	67	39	20	13	8	0
