# Supplementary material for: Organ-specific metastatic landscape dissects PD-(L)1 blockade efficacy in advanced non-small cell lung cancer: applicability from clinical trials to real-world practice
Source: BMC Med. 2022 Apr 12;20:120. doi: 10.1186/s12916-022-02315-2 (PMC9004108; doi:10.1186/s12916-022-02315-2)
Supplement: Supplementary file 9 — Additional file 9: Figure S6. Discrimination performance of METscore for combined immunochemotherapy and bevacizumab in the NFyy cohort. (A) Kaplan-Meier curve of overall survival according to METscore groups (≥ 3 vs. < 3) in patients receiving ICIs plus bevacizumab and chemotherapy. Kaplan-Meier curves of (B) overall survival and (C) progression-free survival according to METscore groups in patients receiving ICIs plus bevacizumab and chemotherapy as first-line treatment. Abbreviations: ICI, immune checkpoint inhibitor; HR, hazard ratio; CI, confidence interval; PD-(L)1, programmed death-(ligand) 1. [file 12916_2022_2315_MOESM9_ESM.pdf]

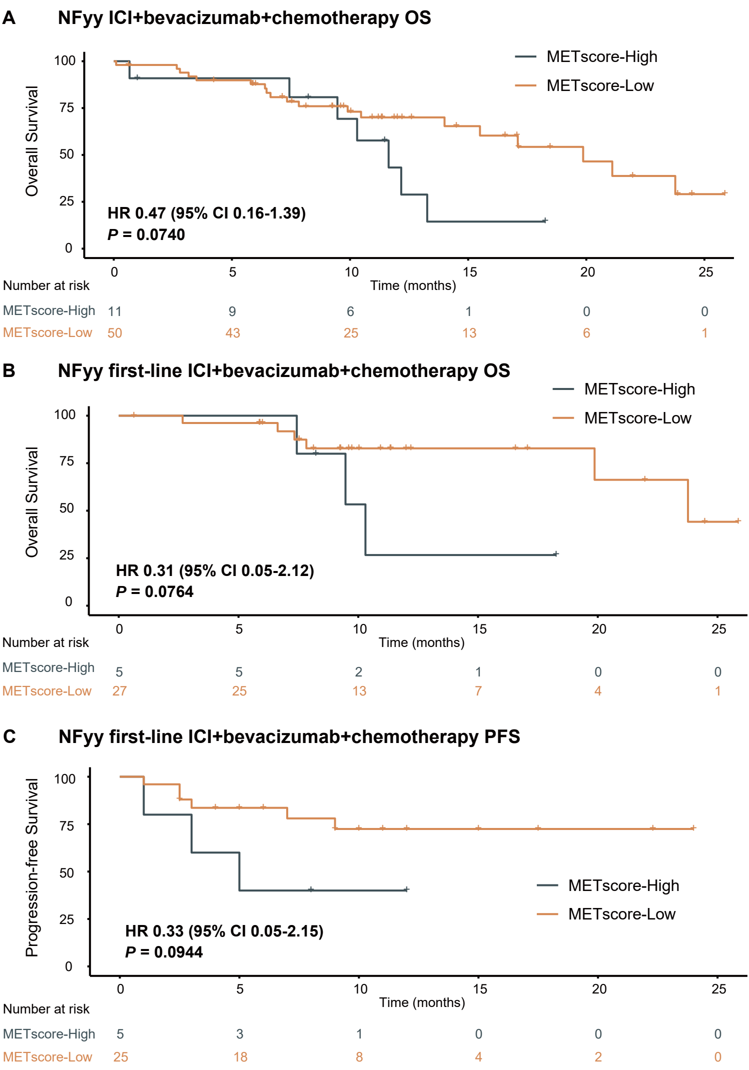
A
NFyy ICI+bevacizumab+chemotherapy OS
METscore-High
METscore-Low
Overall Survival
100
75
50
25
0
HR 0.47 (95% CI 0.16-1.39)
P = 0.0740
0
5
10
15
20
25
Time (months)
Number at risk
METscore-High 11 9 6 1 0 0
METscore-Low 50 43 25 13 6 1
B
NFyy first-line ICI+bevacizumab+chemotherapy OS
METscore-High
METscore-Low
Overall Survival
100
75
50
25
0
HR 0.31 (95% CI 0.05-2.12)
P = 0.0764
0
5
10
15
20
25
Time (months)
Number at risk
METscore-High 5 5 2 1 0 0
METscore-Low 27 25 13 7 4 1
C
NFyy first-line ICI+bevacizumab+chemotherapy PFS
Progression-free Survival
100
75
50
25
0
METscore-High
METscore-Low
HR 0.33 (95% CI 0.05-2.15)
P = 0.0944
0
5
10
15
20
25
Time (months)
Number at risk
METscore-High 5 3 1 0 0 0
METscore-Low 25 18 8 4 2 0
