# Supplementary material for: Organ-specific metastatic landscape dissects PD-(L)1 blockade efficacy in advanced non-small cell lung cancer: applicability from clinical trials to real-world practice
Source: BMC Med. 2022 Apr 12;20:120. doi: 10.1186/s12916-022-02315-2 (PMC9004108; doi:10.1186/s12916-022-02315-2)
Supplement: Supplementary file 6 — Additional file 6: Figure S3. A web-based tool for METscore calculation. (A) An illustration of the use of the web server (https://metscore-ici.github.io/Pages/) for calculating METscore for ICI-treated NSCLC patients and (B) Four examples. Abbreviations: ICI, immune checkpoint inhibitor; NSCLC, non-small cell lung cancer. [file 12916_2022_2315_MOESM6_ESM.pdf]

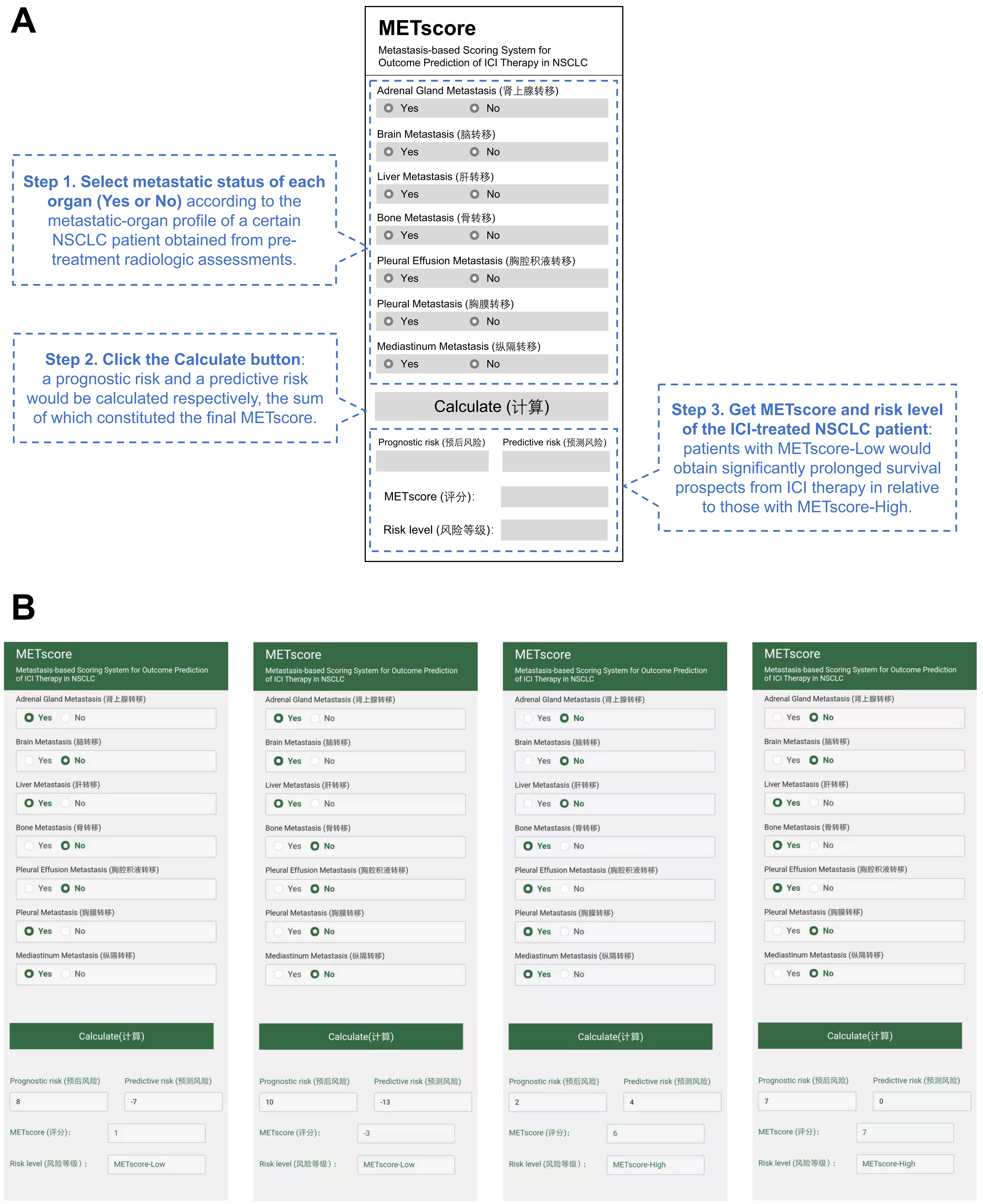
A
METscore
Metastasis-based Scoring System for Outcome Prediction of ICI Therapy in NSCLC
Adrenal Gland Metastasis (肾上腺转移)
Brain Metastasis (脑转移)
Liver Metastasis (肝转移)
Bone Metastasis (骨转移)
Pleural Effusion Metastasis (胸腔积液转移)
Pleural Metastasis (胸膜转移)
Mediastinum Metastasis (纵隔转移)
Yes
No
Calculate (计算)
Prognostic risk (预后风险)
Predictive risk (预测风险)
METscore (评分):
Risk level (风险等级):
Step 1. Select metastatic status of each organ (Yes or No) according to the metastatic-organ profile of a certain NSCLC patient obtained from pre-treatment radiologic assessments.
Step 2. Click the Calculate button: a prognostic risk and a predictive risk would be calculated respectively, the sum of which constituted the final METscore.
Step 3. Get METscore and risk level of the ICI-treated NSCLC patient: patients with METscore-Low would obtain significantly prolonged survival prospects from ICI therapy in relative to those with METscore-High.
B
METscore
Metastasis-based Scoring System for Outcome Prediction of ICI Therapy in NSCLC
Calculate(计算)
Prognostic risk (预后风险)
Predictive risk (预测风险)
METscore (评分):
Risk level (风险等级)：
8
-7
1
METscore-Low
10
-13
-3
METscore-Low
2
4
6
METscore-High
7
0
7
METscore-High
